# Fatigue-related impairments in oculomotor control are prevented by norepinephrine-dopamine reuptake inhibition

**DOI:** 10.1038/srep42726

**Published:** 2017-02-15

**Authors:** Charlotte J. W. Connell, Benjamin Thompson, Jason Turuwhenua, Alexa Srzich, Nicholas Gant

**Affiliations:** 1 Department of Exercise Sciences, Centre for Brain Research, University of Auckland, Auckland, 1142, New Zealand; 2 School of Optometry and Vision Science, University of Waterloo, Ontario, N2L 3G1, Canada; 3 Department of Optometry and Vision Science, University of Auckland, Auckland, 1142, New Zealand

## Abstract

Fatigue-induced reductions in saccade velocity have been reported following acute, prolonged exercise. Interestingly, the detrimental impact of fatigue on oculomotor control can be prevented by a moderate dose of caffeine. This effect may be related to central catecholamine upregulation via caffeine’s action as an adenosine antagonist. To test this hypothesis, we compared the protective effect of caffeine on oculomotor control post-exercise to that of a norepinephrine-dopamine reuptake inhibitor. Within a placebo-controlled crossover design, 12 cyclists consumed placebo, caffeine or a norepinephrine-dopamine reuptake inhibitor (bupropion) during 180 minutes of stationary cycling. Saccades, smooth pursuit and optokinetic nystagmus were measured using infrared oculography. Exercise fatigue was associated with an 8 ± 11% reduction in the peak velocity of prosaccades, and a 10 ± 11% decrement in antisaccade peak velocity. Optokinetic nystagmus quick phases decreased in velocity by 15 ± 17%. These differences were statistically significant (p < 0.05). Norepinephrine-dopamine reuptake inhibition and caffeine prevented fatigue-related decrements in eye movement velocity. Pursuit eye movements and visual attention were unaffected. These findings show that norepinephrine-dopamine reuptake inhibition protects oculomotor function during exercise fatigue. Caffeine’s fatigue-reversing effects on eye movements appear to be mediated, at least in part, via modulation of central catecholamines.

Saccades are rapid changes in fixation that align the foveae with salient targets in the visual field^1^. In a recent study, we reported that saccade velocity is reduced following prolonged exercise and that this effect can be prevented by the administration of caffeine^2^. This suggests that caffeine exerts a protective effect on oculomotor control during exercise-induced fatigue^2^. Furthermore, the detrimental effect of a corticospinal motor system fatigue on the oculomotor system suggests that aspects of exercise-induced central fatigue are ubiquitous within the brain.

The mechanisms responsible for central fatigue are not well understood; though research involving exercising rodents suggests that prolonged physical activity is associated with widespread alterations in the activity of neurotransmitters such as serotonin, dopamine and noradrenaline^3^,^4^,^5^. As such, it has been proposed that these monoamines are involved in the development of fatigue during exercise^6^. Contrary to early hypotheses regarding the involvement of serotonin in the development of central fatigue^7^, it appears that serotonin does not act as a key factor^8^. Instead, the central catecholamines dopamine and norepinephrine are thought to play a principal role in central fatigue.

Studies exploring the influence of central catecholamines on human performance report positive effects on exercise capacity (time to exhaustion), time trial performance and subjective effort sense following the enhancement of central catecholaminergic neurotransmission via targeted pharmacological treatments^9^,^10^,^11^,^12^,^13^. Additionally, caffeine, which indirectly upregulates dopamine and accelerates the synthesis and turnover of norepinephrine via adenosine antagonism^14^, is a common ergogenic aid in prolonged exercise^15^,^16^,^17^,^18^. Overall, these findings provide indirect evidence for an involvement of central catecholamines in the development of central fatigue during whole-body, prolonged exercise. However, measures of exercise performance may be susceptible to confounds from peripheral factors, subject motivation and learning effects. Furthermore, while the benefits of caffeine on exercise performance are largely attributed to its effects on the central nervous system, caffeine also has several actions in the periphery that influence the contractile properties of skeletal muscle, such as increased mobilization of intracellular calcium, and inhibition of phosphodiesterases^19^, that may contribute to certain performance enhancements.

Our previous findings in the oculomotor system demonstrated direct, fatigue-related impairments on motor control and showed that these impairments can be prevented by a moderate dose of caffeine^2^. We propose that the protective effects of caffeine on oculomotor control during fatiguing exercise are the result of indirect upregulation of central catecholaminergic neurotransmission. In this study the effects of caffeine were compared to bupropion, a drug that modulates noradrenergic and dopaminergic reuptake inhibition, increasing the concentration of dopamine and norepinephrine in several brain regions^20^,^21^,^22^,^23^. Acute therapeutic doses of bupropion have previously been reported to confer an ergogenic effect during exercise^9^,^10^.

Oculomotor control was investigated by assessing saccades, smooth pursuit eye movements and optokinetic nystagmus (OKN). Like saccades, smooth pursuit eye movements are constantly utilised during normal visual activity, stabilising visual images on the retina as objects of interest move through the visual field^24^,^25^. OKN is an involuntary oculomotor response elicited by large-scale movement of the visual field, resulting in alternating slow tracking movements (slow phase) followed by rapid, resetting eye movements (quick phase), similar to smooth pursuit and saccades, respectively^26^,^27^. The oculomotor control of eye movements is related to the spatial orienting of attention^28^. To quantify any effects of fatigue on attention, covert spatial attention, or the orienting of attention that occurs without an eye movement toward the attended location, was measured using a cueing paradigm^29^.

In this study a three-hour cycling exercise protocol was used to provide a physiological challenge capable of inducing central fatigue. Prolonged cycling has long-lasting effects on the central nervous system, reducing voluntary activation of the knee extensors for up to 45 minutes following exercise cessation^30^ and causing significant perturbations to cerebral energetics^31^,^32^. Saccades, smooth pursuit, optokinetic nystagmus and covert spatial attention were assessed to investigate the influence of caffeine and norepinephrine-dopamine reuptake inhibition (NDRI) on oculomotor control in the context of prolonged, fatiguing exercise. We predicted that caffeine’s ability to preserve oculomotor control following fatiguing exercise is related to its actions on central catecholamines, therefore a similar influence will be exerted by NDRI.

## Experimental Procedures

### Participants

Twelve healthy volunteers (7 females, maximal aerobic capacity 56 ± 6 ml · kg · min^−1^) with a mean age of 25 (20–48) years and body mass of 74 ± 16 kg, participated in the study. Participants provided written informed consent and visited the laboratory on four occasions to take part in a protocol conducted in accordance with the Declaration of Helsinki and approved by the University of Auckland Human Ethics Committee. Based on responses to a health screening questionnaire, participants were free of medical contraindications to exercise, medication influencing central nervous system function, and had normal or corrected to normal vision.

### Experimental design

**Figure 1:**
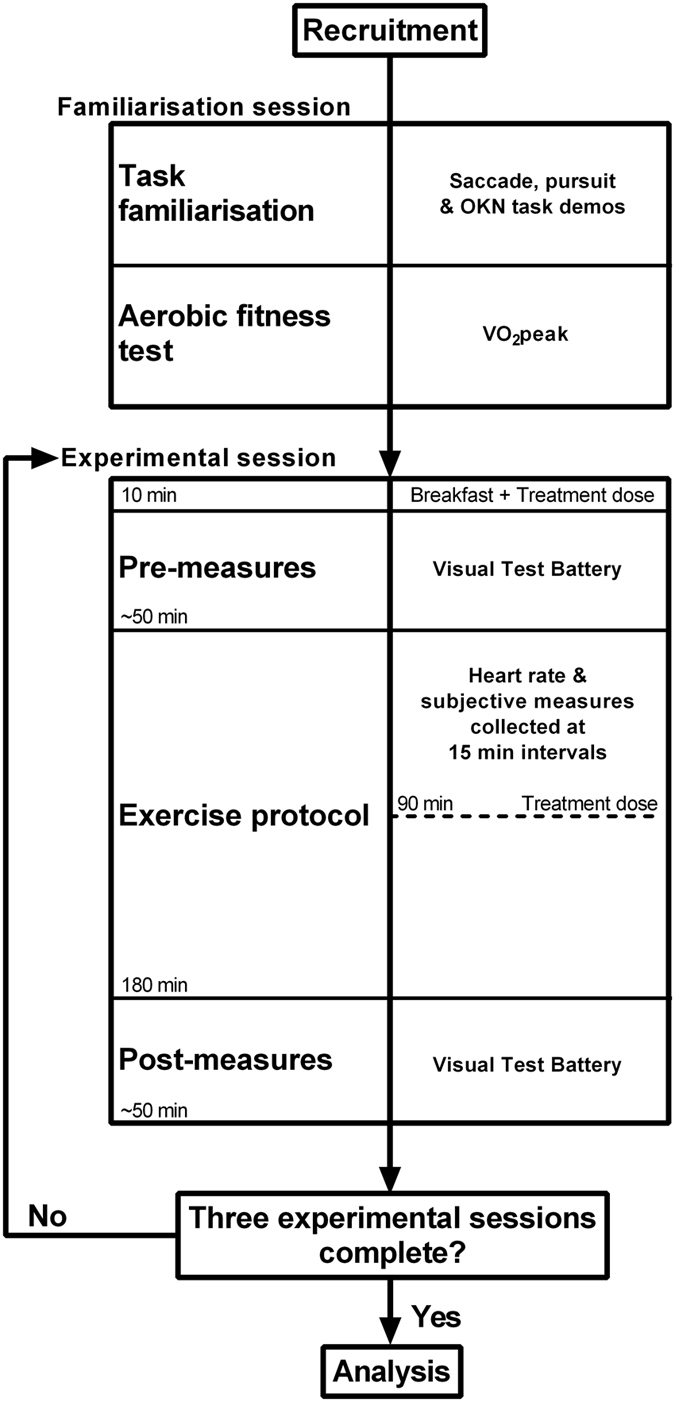
Experimental workflow. Twelve participants were recruited. A familiarisation session accustomed participants with the visual test battery. The three experimental sessions involved completion of the visual test battery at two time points, separated by prolonged cycling exercise lasting 180 minutes. Treatment doses were administered with breakfast, immediately before initial performance of the visual test battery (pre measures), and at 90 minutes through exercise.

Caffeine (7.5 mg · kg^−1^ body mass), bupropion (Zyban, GlaxoSmithKline, Auckland, NZ; maximum therapeutic dose: 300 mg/day) or placebo (maltodextrin) treatments were administered by capsule within a double-blind, placebo-controlled, repeated measures, randomised cross-over design. Participants completed three experimental trials involving 180 minutes of continuous cycling with a self-selected cadence at a work rate equivalent to 60% of maximal aerobic capacity. A minimum of 5 days separated cross-over phases. Caffeine was administered in two doses – a 2.5 mg · kg^−1^ body mass dose 1 hour before the exercise protocol, and a 5 mg · kg^−1^ body mass at 90 minutes into exercise. A 300 mg dose of a NDRI (bupropion) was administered before the exercise protocol, and a placebo pill was given 90 minutes into exercise. In the placebo treatment, a capsule filled with maltodextrin was administered before the exercise protocol and at the 90 minute time point. During exercise, participants were given a carbohydrate solution to maintain hydration and euglycaemia (see *Experimental protocol* below). In all experimental trials, participants completed a battery of visual tasks (see below, *visual performance measures*) before exercise (pre-exercise), and immediately after the exercise protocol (post-exercise). A schematic of the experiment workflow and design is depicted in Fig. 1.

### Preliminary tests

A minimum of one week before the first experimental trial, participants visited the lab for familiarisation with study protocols and the visual test battery tasks (see below, *Visual test battery*). A maximal cardiopulmonary exercise test on an electromagnetically braked cycle ergometer (Velotron Dynafit Pro, Seattle, WA, USA) with respiratory gas analysis equipment (pneumotachometer, MLT1000L, ADInstruments; paramagnetic oxygen analyser, S-3A/I, AEI Technologies; infrared carbon dioxide analyser, CD-3A, AEI Technologies) was performed to measure peak oxygen uptake. Maximal aerobic capacity (VO_2_ max) was estimated and used to prescribe a power output requiring 60% VO_2_ max for the experimental trials.

### Experimental protocol

Participants arrived at the laboratory following a 12 hour overnight fast. Participants were instructed to abstain from caffeine-containing items for 24 hours before each experimental session. Body mass was measured following voiding of the bladder. After this, the first treatment dose was consumed with a breakfast cereal. The quantity of breakfast was self-selected on the first visit and repeated for the remaining trials. Immediately following breakfast, participants completed the visual test battery (~50 minutes). Participants then began 180 minutes of continuous cycling performed in a temperature-controlled exercise chamber maintained at 18° Celsius. At 90 minutes into exercise, participants received a second treatment dose. A carbohydrate solution (0.7 g carbohydrate·kg^−1^ · h^−1^) was ingested every 15-minutes throughout exercise. Mean rates of fluid and carbohydrate ingestion were 645 ± 141 ml · h^−1^ and 52 ± 11 g · h^−1^, respectively. Heart rate was recorded at 15 minute intervals using a heart rate monitor (FS1, Polar Electro, Kempele, Finland). Coincident with heart rate recording, participants’ perceived exertion and felt arousal was self-rated on visual analogue scales. After the exercise protocol, participants completed the visual task battery. A post-exercise measurement of body mass was obtained to assess fluid loss or gain as a result of exercise.

### Visual test battery

The visual test battery consisted of five discrete tasks. Participants were comfortably seated in a quiet, darkened room for the duration of the visual test battery. Stimuli were presented on a cathode ray tube monitor (Philips 109S2; 1280 × 1024 pixel resolution; 85 Hz refresh rate) at a viewing distance of 66 cm. Head movements were minimized using a chin and forehead rest. In four of the tasks, eye movements were tracked with infrared cameras sampling at a rate of 400 Hz (ViewPoint Eye Tracker, Arrington Research Systems, Scottsdale, USA). A 16-point calibration procedure was carried out before the beginning of each task. All participants completed visual tasks in the same order for all experimental sessions. This reduced variance caused by task sequencing effects at the expense of causing the time from exercise cessation to vary across the different tasks.

#### Antisaccade

Each trial began with the presentation of a black, centrally-located circular fixation point subtending a visual angle of 0.5°. A black, circular peripheral target stimulus (0.5° diameter) appeared ± 10° to the left or right of the central fixation point and was visible for a duration of 1000 ms. The peripheral target was presented in *gap* or *overlap* conditions^33^. In the gap condition, the central fixation point was presented for 800 ms and extinguished 200 ms before peripheral target presentation. In the overlap condition, the central fixation point remained visible throughout the trial. Participants were instructed to “look away from the peripheral target stimulus to a mirror opposite position on the screen, and to move your eyes to that position as quickly and accurately as possible”. A total of 100 trials, 50 trials per condition, were presented. The sequence of gap, overlap, right target location and left target location was randomised.

#### Prosaccade

Prosaccade and antisaccade tasks had identical stimulus presentation. However, in the prosaccade task, participants were instead instructed to move their eyes to look at the target stimulus as quickly and accurately as possible.

#### Smooth pursuit

A triangular target waveform was presented at three velocities (5° · s^−1^, 10° · s^−1^ and 30° · s^−1^). The target, a circular black dot (0.8° diameter), was presented in the centre of the screen (0°) for 1000 ms at the start of each trial. Following fixation, the target moved horizontally until it reached ±15°, where it reversed direction abruptly and moved to the opposite side. The direction of the initial ramp was randomised. One trial consisted of 5.5 passes of the target across the display screen. 21 total trials were performed, with each target velocity presented 7 times. The sequence of target velocity and initial ramp direction were randomised. Participants were instructed to follow the target with their eyes as accurately as possible.

#### Optokinetic nystagmus (OKN)

To evoke an OKN response, participants were presented with a stimulus screen consisting of 100% contrast square wave gratings with a fundamental spatial frequency of 0.833 cycles per degree. Gratings were shown for a duration of 20 seconds per trial at two velocities (5° · s^−1^ and 10° · s^−1^) and two stimulus directions (right to left, or left to right) for two trials each, resulting in 8 total trials. A blank, black screen was presented between trials for 4 seconds. Participants were instructed to watch the screen, keeping the stimulus in focus. The objective of these instructions was to encourage a “stare” OKN.

#### Covert spatial attention

A Posner cueing task^29^ with endogenous and exogenous cueing conditions assessed covert attentional orienting. This task has been employed previously to assess the influence of caffeine on covert spatial attention^34^. The start of each trial was marked with a display (1000 ms duration) consisting of a central fixation cross and two peripheral boxes located 10° laterally to the fixation cross. A visual cue was then presented for 200 ms and extinguished. After a further 200 ms, a peripheral target (black circle, radius of 0.5°) appeared in the right or left peripheral box for 150 ms. Participants were instructed to maintain fixation on the central cross and respond as quickly as possible with a key press (left arrow key or right arrow key) when they detected a peripheral target. Two types of cues were presented. *Endogenous cues* consisted of a centrally presented arrow pointing to the left or right peripheral box. Arrows were centred 0.2° above the visual fixation cross and subtended 0.5°. *Exogenous cues* consisted of an increase in the line width of the peripheral box from 0.15° to 0.20°. Cues were presented in valid, invalid or neutral trials. In valid trials the target appeared in the cued location, whilst on invalid trials the peripheral target appeared opposite to the cued location. Neutral trials provided no information regarding the target location. Neutral cues were a doubled-headed arrow, or a change in line width of both peripheral boxes, for endogenous and exogenous conditions, respectively. 90 trials for each condition were presented. In the exogenous condition, valid, invalid and neutral trials occurred with equal probability (30 trials per cue), while for the endogenous condition, the valid cues correctly predicted target location 80 percent of the time. Thus, neutral cues were presented in 30 trials, valid cues in 48 trials and invalid cues in 12 trials. The cue-target contingency for exogenous and endogenous conditions was based on previous literature^29^,^35^,^36^. In both conditions, targets appeared in the right or left peripheral box with equal probability. Cue type was randomised within endogenous and exogenous blocks.

In the preliminary testing session, participants completed abbreviated versions of all visual tasks to ensure they were familiar with stimulus presentation, experimental setup and task requirements.

### Data treatment and analysis

Stimulus presentation, data collection and data analysis were performed using custom software written in Matlab (MathWorks R2010b, Massachusetts, USA). The experimenter visually inspected all eye movement traces. Final sample size satisfied *a priori* power analyses. Sample size was estimated using an expected effect size of 0.4. This effect size was derived from a previous study investigating eye movement kinematics within a similar experimental design^2^. Previous research suggests a high correlation (0.62–0.97) across repeated measures for saccades and smooth pursuit^37^. Thus, with power set to 0.95, p < 0.05, and correlation among repeated measures of 0.62, a sample size of 12 participants was estimated to provide appropriate statistical power for eye movement kinematic measures.

#### Prosaccade and antisaccades

Initiation of a saccade was identified using an amplitude deviation (deviation of >1° from fixation) and a velocity criterion (≥30° · s^−1^). The end of the saccade was detected by a drop in the saccade velocity below 30° · s^−1 ^38,39. Anticipatory saccades (latency <70 ms) were excluded from analysis^40^. Latency (ms), amplitude (°), peak velocity (° · s^−1^) and task performance (percentage of saccades in the correct direction) were the dependent variables derived from the saccade tasks. For kinematic measures, only saccades performed in the correct direction were included in statistical analysis.

#### Smooth pursuits

Saccades and blinks during pursuit were identified by computing a velocity signal from the horizontal eye position during each trial and applying a velocity criterion (≥64° · s^−1^). Sections of pursuit containing saccades and blinks were removed before applying a linear interpolation to the remaining data to calculate eye velocity. Eye velocity was used to derive pursuit gain (eye velocity/target velocity), the dependent measure of the smooth pursuit task. The initial ramp in which the target only completed a pass across half of the screen was not included in analysis.

#### Optokinetic nystagmus

Quick phases were also identified with a velocity criterion (≥64° · s^−1^). Amplitude (°) and peak velocity (° · s^−1^) were derived from the quick phases. Quick phase identification permitted slow phase determination, as slow phases were assumed to occur between quick phases. Slow phase eye velocity was calculated by applying a linear interpolation to the slow phase, thereby allowing the calculation of slow phase gain.

#### Covert spatial attention

Keyboard responses (response time and left/right key) were collected using custom software written in Matlab (Mathworks, R2010b, Massachusetts, USA). A “validity effect” in endogenous and exogenous task conditions for each participant was derived from response times to valid and invalid cues. This measure, which was calculated by subtracting valid response time from invalid response time, reflects the time required to disengage covert attention from an invalidly cued location, and shift attention to the target location. Eye movements were monitored throughout the task. Trials in which the eyes deviated >1° from fixation, or if response time was >1000 ms were rejected from analysis.

### Statistical analyses

Repeated measures analyses of variance (ANOVA) with the factor treatment (Caffeine/NDRI/Placebo) was used to explore the effect of exercise on fluid loss. The validity effect in endogenous and exogenous conditions within the covert spatial attention task was explored by adding the factor timepoint (pre-exercise/post-exercise) to the repeated measures ANOVA. timepoint (pre-exercise/post-exercise) and condition (gap/overlap) were added as factors to statistical analyses for measures from the prosaccade and antisaccade tasks. Similarly, stimulus speed was added as a factor to statistical analyses for smooth pursuit gain (5° · s^−1^, 10° · s^−1^ and 30° · s^−1^) and OKN slow and quick phases (5° · s^−1^, and 10° · s^−1^). There was no influence of stimulus direction, so this was not included as a factor in the statistical analysis. To explore the influence of treatment on subjective experiences and heart rate, the number of levels within timepoint was extended to eleven.

The repeated measures design of the experiment increases possibility of task learning influencing our dependent variables. To identify possible learning effects repeated measures ANOVAs with the factor trial (first vs. second) used in place of treatment. The presence of a task learning effect is commented on where relevant.

Where necessary, interaction effects were explored using within-subject paired comparisons. The multiple comparison type I error rate was controlled using a false discovery rate criterion procedure^41^. Violations of sphericity were controlled with the Greenhouse-Geisser correction. Statistical significance was set at α = 0.05. Results are reported as mean ± standard deviation (SD) unless otherwise stated.

## Results

### Visual performance measures

#### Prosaccades

**Figure 2:**
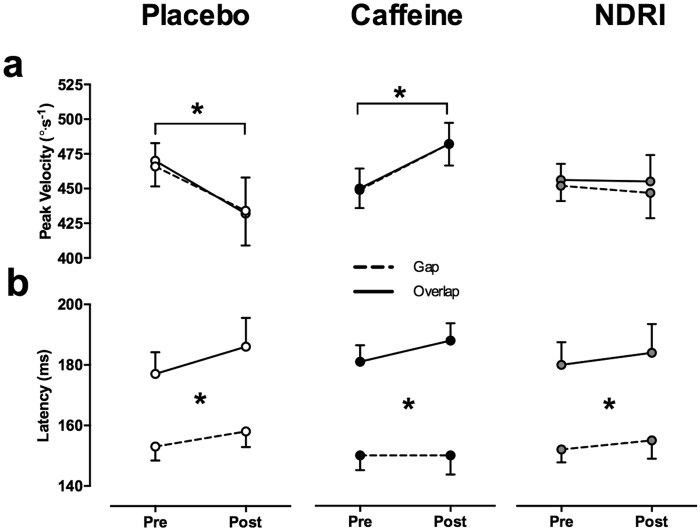
Prosaccade peak velocity and latency for placebo, caffeine and bupropion (NDRI) treatments. (Panel a) – Prosaccade velocity in gap (dashed line) and overlap (solid line) task conditions pre and post exercise. (Panel b) – Prosaccade latency for gap (dashed line) and overlap (solid line) conditions for pre and post exercise categories in placebo, caffeine and NDRI. Here and in Fig. 3, the connecting lines are shown for illustrative purposes only and do not infer the assumption of a linear change from pre to post exercise. Significance labelling between gap and overlap conditions indicates a main effect of condition. Data represent mean ± SE. *<0.05.

**Figure 3:**
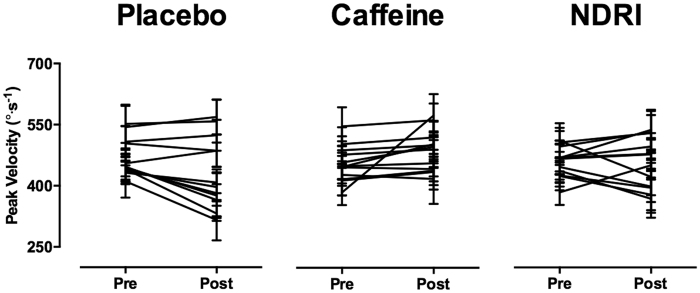
Prosaccade velocity in placebo, caffeine and bupropion (NDRI) treatments for each participant. Peak prosaccade velocity collapsed across task condition for pre and post exercise categories in placebo, caffeine and NDRI treatments. Each point represents mean peak prosaccade velocity ± 95% confidence interval for each participant.

There was an effect of exercise on peak prosaccade velocity that was modulated by treatment (interaction TREATMENT × TIMEPOINT, F_2,22_ = 7.67, p < 0.01). Changes in prosaccade velocity after exercise were similar in magnitude for gap and overlap conditions (Fig. 2, panel a). Post-hoc analysis of peak velocity, collapsed across gap and overlap conditions, revealed that with placebo, exercise-induced fatigue significantly reduced peak velocity compared to pre-exercise by 8 ± 11% (t_23_ = 3.51, p < 0.05). In the majority of participants, prosaccade peak velocity was influenced by exercise-induced fatigue in the placebo trial, with eight of twelve participants exhibiting a lower post-exercise peak prosaccade velocity compared to pre-exercise. To explore the magnitude of this change while accounting for within-subject variability, an effect size comparing pre-exercise prosaccade velocity to post exercise was calculated for each participant. The average effect size of the eight participants that displayed a drop in peak prosaccade velocity with placebo was 0.77 ± 0.41. The remaining participants displayed an increase in peak velocity post-exercise with an average effect size of 0.19 ± 0.13. Conversely, caffeine prevented this exercise-induced decrement in prosaccade velocity and increased velocity significantly above pre-exercise levels (6 ± 9%, t_23_ = −2.96, p < 0.05)(see Fig. 2, panel a). Ten of twelve participants exhibited an increase in prosaccade velocity above pre-exercise levels. However, a large increase was evident in one participant (effect size >1.0), while the remaining nine participants exhibited only a small to moderate increase in velocity post exercise (average effect size, 0.24 ± 0.17). Two participants displayed a drop in prosaccade peak velocity following exercise, however, this was only of a small magnitude (average effect size, 0.09 ± 0.06). NDRI also prevented post exercise decrements in peak prosaccade velocity, maintaining velocity at pre-exercise levels (−1 ± 12%, t_23_ = 0.33, p = 0.74). Seven participants displayed a slight increase in prosaccade velocity post-exercise, with an average effect size of 0.40 ± 0.32, while five participants exhibited a moderate decrease post-exercise (average effect size, 0.66 ± 0.2.4). Figure 3 depicts average peak prosaccade velocity (collapsed across gap and overlap conditions) pre and post-exercise for placebo, caffeine and NDRI treatments for each participant.

A robust main effect of condition was observed for prosaccade latency (F_1,11_ = 45.27, p < 0.01) whereby prosaccades in the overlap condition were performed with slower latencies compared to the gap condition. The influence of condition on prosaccade latency pre and post exercise for each treatment is illustrated in panel b of Fig. 2. No significant changes were observed across time point or between treatments for prosaccade latency. Similarly, condition also influenced task performance, with higher performance in the overlap condition (F_1,11_ = 14.12, p < 0.05). However, task performance in both conditions was modulated by an interaction between treatment and time point (F_2,22_ = 4.11, p < 0.05). Post-hoc analyses revealed a significant drop in task performance post exercise with NDRI (t_23_ = 2.93, p < 0.05), while there was no change in task performance following exercise with caffeine or placebo treatments. There were no alterations in prosaccade amplitude as a result of condition, time point or treatment. These data are reported in Table 1.

#### Antisaccades

There was an effect of exercise-induced fatigue on antisaccade peak velocity that was modulated by treatment and condition (3-way interaction TREATMENT × TIMEPOINT × CONDITION, F_2, 22_ = 7.98, p < 0.01). Post-hoc analysis revealed that exercise-induced fatigue significantly reduced the peak velocity of antisaccades in the gap condition compared to pre-exercise by 10 ± 11%. Nine participants displayed a moderate decrease in antisaccade peak velocity (average effect size, 0.58 ± 0.58) with the placebo. The remaining three participants exhibited a small to moderate increase in peak velocity following exercise after placebo (average effect size, 0.29 ± 0.10). Conversely, peak antisaccade velocity did not differ across time point in the placebo treatment for the overlap condition, nor were any significant alterations in antisaccade peak velocity detected across time and condition for caffeine and NDRI treatments.

A main effect of condition was observed for antisaccade latency (F_1,11_ = 8.39, p < 0.05) whereby antisaccades in the gap condition had faster latencies than the overlap condition. An interaction effect between condition and time point also modulated antisaccade latency (F_1,11_ = 6.11, p < 0.05). This appeared to stem from longer latencies post exercise in the overlap condition, whereas gap latencies remain stable across time. However, the increase in overlap latencies post exercise did not reach statistical significance after correcting for multiple comparisons (t_35_ = 1.71, p = 0.05, critical p < 0.025). A robust effect of condition was detected for task performance, with a higher percentage of antisaccades performed in the correct direction in the overlap condition (91 ± 9%) compared to the gap condition (80 ± 14%)(F_1, 11_ = 26.09, p < 0.01). There were no alterations in antisaccade amplitude as a result of condition, time point or treatment.

#### Smooth pursuit

Pursuit gain differed depending on the stimulus speed presented (F_2,18_ = 95.40, p < 0.01). For the slowest stimulus speed (5° · s^−1^), pursuit gain was closest to unity and progressively declined for faster stimulus speeds (10° · s^−1^ and 30° · s^−1^). An interaction between stimulus speed and time point was detected for pursuit gain (F_2,18_ = 7.78, p < 0.01). Post-hoc tests revealed that this effect stems from a decrease in smooth pursuit gain post-exercise, irrespective of treatment, for the 30° · s^−1^ stimulus speed (t_29_ = 4.69, p < 0.01). These data are reported in Table 1.

### Optokinetic nystagmus

Slow phase gain of OKN was modulated by an interaction between stimulus speed and treatment (F_2,20_ = 4.53, p < 0.05). Post-hoc tests revealed significantly reduced slow phase gain in response to the 10° · s^−1^ stimulus speeds with placebo compared to caffeine (t_21_ = 2.79 p < 0.01) and NDRI (t_21_ = 2.87, p < 0.01) respectively.

Alterations in amplitude and peak velocity of OKN quick phases were detected. Amplitude differed depending on stimulus speed presented (F_1,10_ = 28.93, p < 0.01), with slightly higher quick phase amplitudes in response to the 10° · s^−1^ speed (5.3 ± 1.6°) compared to the 5° · s^−1^ speed (4.3 ± 1.68). Amplitude was also modulated by an interaction between treatment and time point (F_2,20_ = 3.88, p < 0.05). This stemed from a slight increase in amplitude post-exercise with caffeine (pre-exercise, 4.7 ± 1.4°; post-exercise, 5.3 ± 2.0°). However, this difference was not statistically significant after correcting for multiple comparisons.

**Figure 4:**
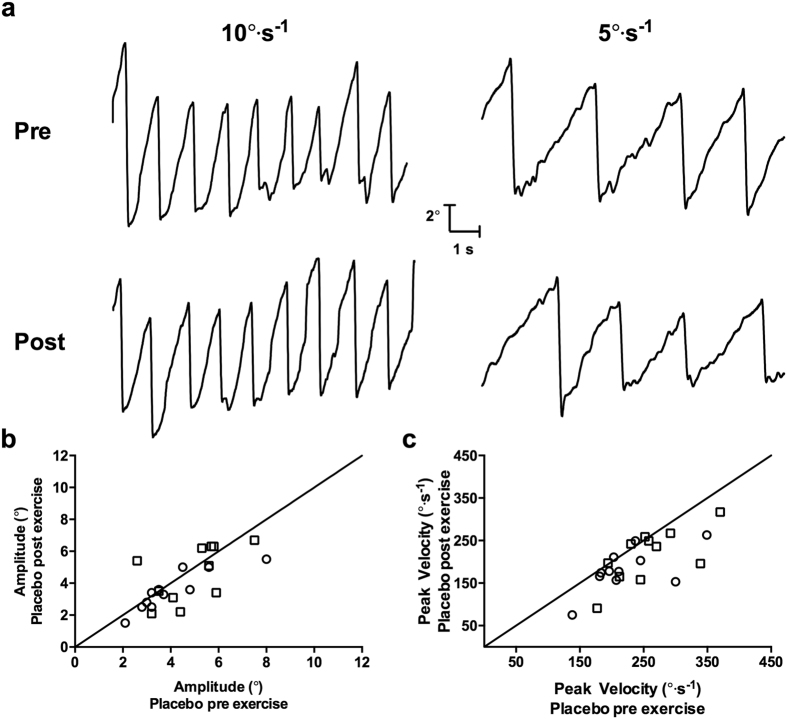
Kinematic characteristics of OKN quick phases in placebo treatment. (Panel a) – Sample eye movement traces of optokinetic nystagmus pre exercise (top traces) and post exercise (bottom traces) in response to 10° · s^−1^ (left traces) and 5° · s^−1^ (right traces) stimulus speeds. (Panel b) – Mean amplitude of OKN quick phases pre and post exercise categories with placebo. Each symbol represents one participant, while the shape of the symbol corresponds to one stimulus velocity: 5° · s^−1^ (circle) and 10° · s^−1^ (square). The diagonal unity line represents no difference between pre exercise and post exercise. (Panel c) – Mean peak velocity of OKN quick phases in placebo and caffeine.

Peak velocity of quick phases also differed between stimulus speeds (F_1,10_ = 23.74, p < 0.01), with higher velocities to the 10° · s^−1^ (249 ± 57° · s^−1^) compared to the 5° · s^−1^ per second stimulus speed (134 ± 28° · s^−1^). An interaction between treatment and time point was also evident (F_2,20_ = 7.51, p < 0.01). Post-hoc tests revealed a significant decrease in peak velocity post-exercise with placebo (t_21_ = 3.80, p < 0.01), while there were no differences pre to post-exercise in peak velocity with caffeine and NDRI treatments. These data, along with example OKN traces pre and post-exercise with placebo are illustrated in Fig. 4.

#### Covert spatial attention

The number of trials rejected due to deviations from fixation did not differ as a consequence of treatment or time point. The validity effect in endogenous and exogenous cueing conditions was equivalent between treatments and across time points. Table 2 reports the validity effect for both task conditions across time for each treatment.

### Heart rate, fluid balance and subjective measures

**Figure 5:**
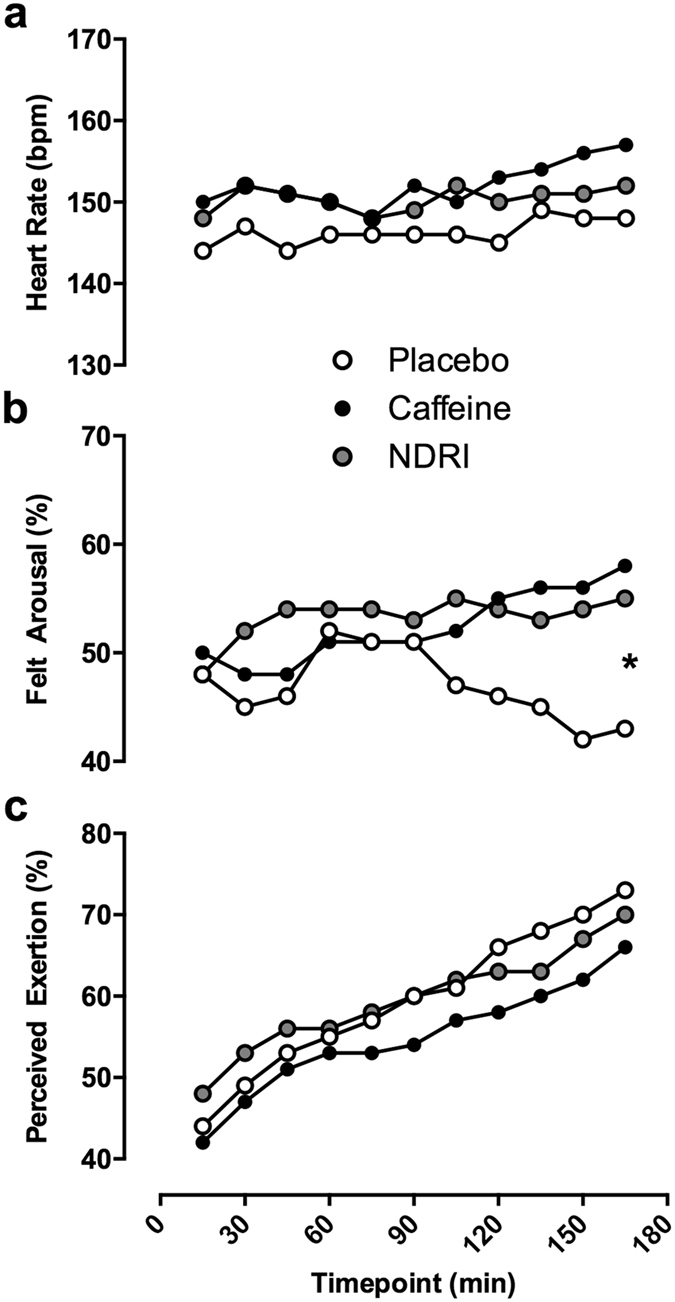
Heart rate, felt arousal and perceived exertion during exercise for placebo (white fill) caffeine (black fill) and NDRI (gray fill). (Panel a) – Heart rate across the 180 minute exercise protocol in experimental treatments. (Panel b) – Felt arousal across the 180 minute exercise protocol. Significance labelling represents a significant posthoc comparison between placebo versus caffeine and NDRI at the 165 minute time point through exercise. (Panel c) – Perceived exertion across the 180 minute exercise protocol. Standard error for each point is not depicted here to improve the clarity of the figure. *<0.05.

Heart rate and fluid loss were equivalent between treatments. For placebo, caffeine and NDRI treatments average heart rate (beats per minute) was 147 ± 19, 150 ± 18, and 153 ± 20 respectively. Figure 5 (panel a) illustrates heart rate over time for each treatment. Mean change in body mass was −0.2 ± 0.7%, −0.1 ± 0.8% and −0.3% ± 0.9% of pre-exercise body mass, for placebo, caffeine and NDRI treatments respectively. There was no influence of treatment on change in body mass (F_2,22_ = 1.00, p = 0.40).

Figure 5 (panels b and c) shows average ratings of perceived exertion and felt arousal for placebo, caffeine and NDRI treatments. A main effect of time (F_10,110_ = 20.01, p < 0.05) was detected for ratings of perceived exertion. Perceived exertion steadily increased over the duration of the exercise protocol, to a similar extent for all treatments.

An interaction between treatment and time (F_20,220_ = 2.50, p < 0.05) was found for felt arousal. Felt arousal with NDRI appears relatively stable across the duration of the exercise protocol, and was higher than caffeine and placebo for the first 90 minutes of exercise. After 90 minutes, felt arousal with caffeine started to increase, reaching a maximum by 165 minutes into exercise. Conversely, after 90 minutes, felt arousal in placebo steadily declined. Post-hoc comparisons revealed a significant difference in felt arousal at the 165 minutes between caffeine and placebo (t_11_ = 3.60, p < 0.05), and NDRI and placebo (t_11_ = 2.20, p < 0.05), while there was no difference between caffeine and NDRI (t_11_ = 1.01, p = 0.33).

## Discussion

This study provides evidence for a role of noradrenergic and dopaminergic neurotransmission in fatigue-related impairments in the oculomotor control of high-velocity eye movements. The findings suggest that caffeine exerts at least part of its protective effects through these neurochemicals. Furthermore, the detrimental effects of fatigue on eye movement kinematics were restricted to velocity, while eye movement amplitude, tracking performance of pursuit eye movements and visual attention were robust to fatigue and psychotropic drugs.

In a previous study^2^, we reported a significant decrease in average prosaccade velocity following prolonged exercise that could not be accounted for by changes in saccade amplitude. This effect was proposed to result from central fatigue. In the present study, using measures of peak eye movement velocity, we extended these findings by observing that prolonged exercise exerts a similar effect on the velocities of pro- and antisaccades as well as the quick phase of OKN. This suggests that the detrimental influence of fatigue on saccade velocity is stable across differing task conditions and even extends to reflexive eye movements. Whether the velocity reductions we measured (8–10% reduction in peak velocity) have a discernible influence on visual function is yet to be determined. However, it is plausible that fatigue-related reductions in saccade velocity lengthen the time required to gather information from the visual field.

The detrimental effect of exercise-induced fatigue did not extend to smooth pursuit or the slow phase of OKN, as gain was not modulated as a result of exercise or treatment. The slow phase gain of OKN for the 10° · s^−1^ stimulus was significantly lower in the placebo condition than the treatment conditions. This may reflect a pre-exercise influence of caffeine and norepinephrine-dopamine reuptake inhibition as participants received treatment doses before starting the pre-exercise visual test battery.

Caffeine and norepinephrine-dopamine reuptake inhibition exerted a similar effect on oculomotor control, protecting the peak velocity of saccades and the quick phases of OKN following exercise. Both treatments increase levels of norepinephrine and dopamine release^20^,^42^. Therefore, we propose that prolonged exercise impaired the synthesis and metabolism of central catecholamines resulting in inhibitory tone that disrupted oculomotor function. This effect appeared to influence both subcortical and cortical components of the oculomotor control system. In particular, exercise fatigue affected reflexive eye movements (prosaccades, OKN quick-phase) that involve the paramedian pontine formation (PPRF), superior colliculus and basal ganglia^43^,^44^,^45^ as well as antisaccades that also involve frontal cortical oculomotor regions such as the frontal eye fields, supplementary eye fields and dorsolateral prefrontal cortex^45^. The effects may have been most pronounced for regions within the oculomotor network, such as the PPRF burst neurons, that generate the neural drive for high-velocity eye movements and that utilize dopamine and norepinephrine^46^, ^47^, ^48^.

The measures of visual attention derived from the gap-overlap paradigm within the saccade tasks and the covert spatial attention task assessed the influence of exercise-induced fatigue on attentional factors involved in oculomotor control. A clear effect of gap and overlap conditions, similar in magnitude, was observed for saccade latency and task performance across all experimental treatments. In prosaccades and antisaccades, latencies in the gap condition were significantly shorter than those in the overlap condition (Fig. 2 and Table 1, respectively). Primate research has shown that gap trials are associated with higher preparatory neural activity in the frontal eye fields, the superior colliculus and the paramedian pontine reticular formation^49^,^50^,^51^, and inhibition of fixation-related activity in the superior colliculus^50^. This pattern of neural activity is thought to promote a reduction in saccadic latency and lower task performance rates in gap trials^52^,^53^. Conversely, in the overlap trials, persistence of the fixation point during onset of the peripheral target promotes fixation-related neural activity after target appearance, causing slower saccade latencies^54^. Thus, it appears that exercise-induced fatigue does not selectively influence the release or maintenance of visual fixation, nor does it exert a general effect on the mechanisms underlying the disengagement and re-engagement of visual attention. Further, the orienting of covert spatial attention was unaffected by exercise-fatigue. Overall, these findings suggest that components of visual attention relevant to oculomotor control are not vulnerable to the central effects of fatigue, and are unlikely to have contributed to the alterations in eye movement kinematics that were observed following exercise.

To our knowledge, this is the first study to compare the actions of caffeine and norepinephrine-dopamine reuptake inhibition during fatigue. Both treatments prevented fatigue-related alterations in oculomotor control with caffeine exerting a slightly larger effect. It is likely that the more pronounced effect of caffeine was a consequence of a dose discrepancy between the two treatments as it was not possible to possible to titrate caffeine and bupropion doses to equivalent influence on catecholaminergic neurotransmission.

Because we imposed equivalent physiological stress between trials, within a protocol that elicits perturbations in cerebral energetics^55^ we are able to contrast the influence of treatments on arousal during exercise, which has not been reported previously with these drugs. Caffeine and NDRI exerted a similar effect on subjective ratings of arousal, promoting significantly higher levels of arousal for 165 minutes of exercise compared to placebo. The observed effects on arousal confirm adequate dosing as this response is consistent with previous reports of the influence of caffeine during exercise^56^.

One of the strengths of this study is that it averted some of the difficulties of investigating alterations in central nervous system function following whole-body exercise by measuring motor output from the relatively well understood neural circuitry of the human oculomotor system^25^,^45^,^57^,^58^. However, the interpretation of our findings is somewhat limited to prolonged steady-state exercise. Further evaluation is required to establish the manner in which whole-body exercise of differing modes, intensities and durations influences the development of central fatigue detectable by changes in oculomotor control.

Overall, our findings suggest that caffeine’s protective effect on oculomotor control following exercise-induced fatigue is mediated by its action on central catecholaminergic neurotransmission, as targeted manipulation of these neurotransmitter systems via norepinephrine-dopamine reuptake inhibition also prevented fatigue-related impairments to the peak velocity of quick phases and saccades. These observations cannot be explained by direct stress to the brain circuitry controlling the extra-ocular muscles as the oculomotor system is functionally independent of the locomotion, and was not challenged during cycling exercise.

## Additional Information

**How to cite this article:** Connell, C. J. W. *et al*. Fatigue-related impairments in oculomotor control are prevented by norepinephrine-dopamine reuptake inhibition. *Sci. Rep.* **7**, 42726; doi: 10.1038/srep42726 (2017).

**Publisher's note:** Springer Nature remains neutral with regard to jurisdictional claims in published maps and institutional affiliations.

## Figures and Tables

**Table 1:** Measures of oculomotor control.

	Placebo				Caffeine				Bupropion			
	Pre exercise		Post exercise		Pre exercise		Post exercise		Pre exercise		Post exercise	
	Mean	SD	Mean	SD	Mean	SD	Mean	SD	Mean	SD	Mean	SD
**Prosaccade**												
***Peak velocity*****(° · *****s***^**−*****1***^)	***468***	***47***	***433***	***88***	***454***	***44***	***485***	***50***	***458***	***37***	***455***	***62***
*Gap condition*	466	50	434	87	453	44	485	52	456	37	451	61
*Overlap condition*	470	44	432	90	454	48	485	51	460	39	459	64
***Latency**(**ms***)	***167***	***18***	***174***	***26***	***166***	***17***	***171***	***16***	***167***	***18***	***170***	***26***
*Gap condition*	155	14	160	18	151	16	153	18	154	14	155	20
*Overlap condition*	179	26	187	34	182	20	189	19	181	25	185	33
***Amplitude*****(°)**	***9***	***0.5***	***9***	***0.9***	***9***	***0.4***	***9***	***0.6***	***9***	***0.6***	***9***	***0.9***
*Gap condition*	9	0.6	9	0.5	9	0.6	9	0.7	9	0.6	9	0.9
*Overlap condition*	9	0.4	9	1.5	9	0.4	9	0.6	9	0.7	9	0.9
***Task performance*****(%*****correct***)	***97***	***4***	***96***	***2***	***96***	***5***	***97***	***5***	***98***	***3***	***95***	***5***
*Gap condition*	95	6	96	4	92	9	95	8	95	5	92	8
*Overlap condition*	99	3	100	1	99	1	99	2	100	1	98	3
**Antisaccade**												
***Peak velocity*****(° · *****s***^**−*****1***^)	***444***	***62***	***413***	***63***	***441***	***73***	***462***	***82***	***432***	***40***	***413***	***62***
*Gap condition*	457	61	411	70	443	76	465	84	433	41	421	66
*Overlap condition*	432	66	416	58	439	70	458	81	431	43	404	61
***Latency**(**ms***)	***231***	***61***	***247***	***43***	***238***	***38***	***233***	***37***	***242***	***45***	***242***	***52***
*Gap condition*	228	63	239	47	232	41	225	43	240	49	236	56
*Overlap condition*	233	60	254	40	243	35	241	32	244	42	248	50
***Amplitude**(**°***)	***9***	***2.1***	***9***	***1***	***9***	***1.8***	***9***	***1.6***	***9***	***1.8***	***9***	***1.5***
*Gap condition*	9	2.1	9	1	9	1.8	9	1.6	9	1.7	9	1.4
*Overlap condition*	9	2.1	9	1	9	1.9	9	1.6	9	2	9	1.7
***Task performance**(**% correct***)	***82***	***17***	***87***	***8***	***87***	***7***	***87***	***8***	***88***	***10***	***85***	***11***
*Gap condition*	76	18	82	12	83	11	80	13	82	15	78	14
*Overlap condition*	87	18	91	5	92	4	94	4	93	7	92	8
**Smooth pursuit**												
***Gain***	***0.91***	***0.26***	***0.87***	***0.29***	***0.89***	***0.26***	***0.87***	***0.3***	***0.87***	***0.23***	***0.9***	***0.31***
*5*° · *s*^−*1*^ *stimulus*	0.97	0.27	0.96	0.26	0.96	0.25	0.94	0.33	0.93	0.23	0.96	0.29
*10*° · *s*^−*1*^ *stimulus*	0.91	0.23	0.91	0.27	0.9	0.25	0.86	0.26	0.89	0.23	0.94	0.32
*30*° · *s*^−*1*^ *stimulus*	0.75	0.19	0.66	0.25	0.73	0.22	0.7	0.22	0.74	0.17	0.67	0.23
**Optokinetic Nystagmus**												
***Slow phase gain***	***0.67***	***0.11***	***0.64***	***0.11***	***0.74***	***0.08***	***0.72***	***0.07***	***0.7***	***0.1***	***0.73***	***0.1***
*5*° · *s*^−*1*^ *stimulus*	0.72	0.1	0.65	0.06	0.71	0.06	0.7	0.07	0.67	0.07	0.7	0.09
*10*° · *s*^−*1*^ *stimulus*	0.64	0.21	0.65	0.16	0.76	0.14	0.74	0.1	0.72	0.16	0.75	0.14
***Quick phase amplitude**(**°***)	***5***	***1.4***	***4***	***1.5***	***5***	***1.2***	***5***	***1.9***	***5***	***1.5***	***5***	***1.8***
*5*° · *s*^−*1*^ *stimulus*	4	1.7	4	1.2	4	1.3	5	2.2	4	1.8	4	1.6
*10*° · *s*^−*1*^ *stimulus*	5	1.4	5	1.8	5	1.3	6	1.9	6	1.4	6	2
***Quick phase peak velocity*****(° · *****s***^**−*****1***^)	***245***	***54***	***203***	***54***	***235***	***52***	***245***	***58***	***250***	***47***	***237***	***62***
*5*° · *s*^−*1*^ *stimulus*	223	59	182	51	213	52	232	71	218	23	208	57
*10*° · *s*^−*1*^ *stimulus*	258	59	216	63	248	53	254	54	266	44	254	66

Kinematic and task performance measures derived from prosaccade, antisaccade, smooth pursuit and OKN visual tasks, pre and post exercise for placebo, caffeine and NDRI treatments.

**Table 2:** Measures of covert spatial attention.

	Placebo				Caffeine				NDRI			
	Pre exercise		Post exercise		Pre exercise		Post exercise		Pre exercise		Post exercise	
	Mean	SD	Mean	SD	Mean	SD	Mean	SD	Mean	SD	Mean	SD
Validity effect												
*Endogenous (ms*)	48	33	58	46	42	36	58	45	42	35	61	51
*Exogenous (ms*)	25	29	28	37	18	25	37	30	18	27	13	22

Validity effect for endogenous and exogenous conditions of the covert spatial attention task across placebo, caffeine and NDRI treatments.

## References

[b1] Leigh R. J. & Kennard C. Using saccades as a research tool in the clinical neurosciences. Brain 127, 460–477 (2004). 14607787 10.1093/brain/awh035

[b2] Connell C. J. W. . Fatigue related impairments in oculomotor control are prevented by caffeine. Sci. Rep. 6, 26614 (2016). 27222342 10.1038/srep26614 PMC4879569

[b3] Foley T. E. & Fleshner M. Neuroplasticity of dopamine circuits after exercise: implications for central fatigue. Neuromolecular Med. 10, 67–80 (2008). 18274707 10.1007/s12017-008-8032-3

[b4] Meeusen R. . Effects of tryptophan and/or acute running on extracellular 5-HT and 5-HIAA levels in the hippocampus of food-deprived rats. Brain Res. 740, 245–252 (1996). 8973821 10.1016/s0006-8993(96)00872-4

[b5] Meeusen R. . Endurance training effects on neurotransmitter release in rat striatum: an *in vivo* microdialysis study. Acta Physiol. Scand. 159, 335–341 (1997). 9146755 10.1046/j.1365-201X.1997.00118.x

[b6] Meeusen R., Watson P., Hasegawa H., Roelands B. & Piacentini M. F. Central fatigue: the serotonin hypothesis and beyond. Sports Med. 36, 881–909 (2006). 17004850 10.2165/00007256-200636100-00006

[b7] Newsholme E., Acworth I. & Blomstrand E. Amino acids, brain neurotransmitters and a functional link between muscle and brain that is important in sustained exercise. Adv. Myochem. 1, 127–133 (1987).

[b8] Taylor J. L., Amann M., Duchateau J., Meeusen R. & Rice C. L. Neural contributions to muscle fatigue: from the brain to the muscle and back again. Med. Sci. Sports Exerc (2016). 10.1249/MSS.0000000000000923 PMC5033663 27003703

[b9] Watson P. . Acute dopamine/noradrenaline reuptake inhibition enhances human exercise performance in warm, but not temperate conditions. J. Physiol. 565, 873–883 (2005). 15831540 10.1113/jphysiol.2004.079202 PMC1464564

[b10] Roelands B. . A dopamine/noradrenaline reuptake inhibitor improves performance in the heat, but only at the maximum therapeutic dose. Scand. J. Med. Sci. Sports 22, e93–e98 (2012). 22845895 10.1111/j.1600-0838.2012.01502.x

[b11] Piacentini M. F., Meeusen R., Buyse L., De Schutter G. & De Meirleir K. Hormonal responses during prolonged exercise are influenced by a selective DA/NA reuptake inhibitor. Br. J. Sports Med. 38, 129–133 (2004). 15039245 10.1136/bjsm.2002.000760 PMC1724779

[b12] Jacobs I. & Bell D. G. Effects of acute modafinil ingestion on exercise time to exhaustion. Med. Sci. Sports Exerc. 36, 1078–1082 (2004). 15179180 10.1249/01.mss.0000128146.12004.4f

[b13] Swart J. . Exercising with reserve: evidence that the central nervous system regulates prolonged exercise performance. Br. J. Sports Med. 43, 782–788 (2009). 19052141 10.1136/bjsm.2008.055889

[b14] Kalmar J. M. & Cafarelli E. Caffeine: a valuable tool to study central fatigue in humans? Exerc. Sport Sci. Rev. 32, 143–147 (2004). 15604932 10.1097/00003677-200410000-00004

[b15] Cox G. R. . Effect of different protocols of caffeine intake on metabolism and endurance performance. J. Appl. Physiol. 93, 990–999 (2002). 12183495 10.1152/japplphysiol.00249.2002

[b16] Pasman W. J., van Baak M. A., Jeukendrup A. E. & de Haan A. The effect of different dosages of caffeine on endurance performance time. Int. J. Sports Med. 16, 225–230 (1995). 7657415 10.1055/s-2007-972996

[b17] Bell D. G. & McLellan T. M. Exercise endurance 1, 3, and 6 h after caffeine ingestion in caffeine users and nonusers. J. Appl. Physiol. 93, 1227–1234 (2002). 12235019 10.1152/japplphysiol.00187.2002

[b18] Anderson M. E. . Improved 2000-meter rowing performance in competitive oarswomen after caffeine ingestion. Int. J. Sport Nutr. Exerc. Metab. 10, 464–475 (2000). 11099373 10.1123/ijsnem.10.4.464

[b19] Nehlig A., Daval J. L. & Debry G. Caffeine and the central nervous system: mechanisms of action, biochemical, metabolic and psychostimulant effects. Brain Res. Rev. 17, 139–170 (1992). 1356551 10.1016/0165-0173(92)90012-b

[b20] Hasegawa H. . Acute dopamine/norepinephrine reuptake inhibition increases brain and core temperature in rats. J. Appl. Physiol. 99, 1397–1401 (2005). 15920099 10.1152/japplphysiol.00435.2005

[b21] Sidhpura N., Redfern P., Rowley H., Heal D. & Wonnacott S. Comparison of the effects of bupropion and nicotine on locomotor activation and dopamine release *in vivo*. Biochem. Pharmacol. 74, 1292–1298 (2007). 17678630 10.1016/j.bcp.2007.06.025

[b22] Piacentini M. F. . Effect of bupropion on hippocampal neurotransmitters and on peripheral hormonal concentrations in the rat. J. Appl. Physiol. 95, 652–656 (2003). 12692144 10.1152/japplphysiol.01058.2002

[b23] Bredeloux P., Dubuc I. & Costentin J. Comparisons between bupropion and dexamphetamine in a range of *in vivo* tests exploring dopaminergic transmission. Br. J. Pharmacol. 150, 711–719 (2007). 17293887 10.1038/sj.bjp.0707151 PMC2013864

[b24] Nobre A. C., Gitelman D. R., Dias E. C. & Mesulam M. M. Covert visual spatial orienting and saccades: overlapping neural systems. Neuroimage 11, 210–216 (2000). 10694463 10.1006/nimg.2000.0539

[b25] Posner M. I. Orienting of attention. Q. J. Exp. Psychol. 32, 3–25 (1980). 7367577 10.1080/00335558008248231

[b26] Bense S. . Brainstem and cerebellar fMRI-activation during horizontal and vertical optokinetic stimulation. Exp. Brain Res. 174, 312–323 (2006). 16636788 10.1007/s00221-006-0464-0

[b27] Garbutt S., Harwood M. & Harris C. Comparison of the main sequence of reflexive saccades and the quick phases of optokinetic nystagmus. Br. J. Ophthalmol. 85, 1477–1483 (2001). 11734524 10.1136/bjo.85.12.1477 PMC1723810

[b28] Sidhu S. K., Bentley D. J. & Carroll T. J. Locomotor exercise induces long-lasting impairments in the capacity of the human motor cortex to voluntarily activate knee extensor muscles. J. Appl. Physiol. 106, 556–565 (2009). 19056999 10.1152/japplphysiol.90911.2008

[b29] Nybo L., Nielsen B., Blomstrand E., Møller K. & Secher N. Neurohumoral responses during prolonged exercise in humans. J. Appl. Physiol. 95, 1125–1131 (2003). 12754171 10.1152/japplphysiol.00241.2003

[b30] Nybo L., Dalsgaard M. K., Steensberg A., Møller K. & Secher N. H. Cerebral ammonia uptake and accumulation during prolonged exercise in humans. J. Physiol. 563, 285–290 (2005). 15611036 10.1113/jphysiol.2004.075838 PMC1665558

[b31] Fischer B., Gezeck S. & Hartnegg K. The analysis of saccadic eye movements from gap and overlap paradigms. Brain Res. Protoc. 2, 47–52 (1997). 10.1016/s1385-299x(97)00027-5 9438071

[b32] Connell C. J. W., Thompson B., Kuhn G. & Gant N. Exercise-induced fatigue and caffeine supplementation affect psychomotor performance but not covert visuo-spatial attention. PLoS ONE 11, e0165318, doi: 10.1371/journal.pone.0165318 (2016). 27768747 PMC5074788

[b33] Peelen M. V., Heslenfeld D. J. & Theeuwes J. Endogenous and exogenous attention shifts are mediated by the same large-scale neural network. Neuroimage 22, 822–830 (2004). 15193611 10.1016/j.neuroimage.2004.01.044

[b34] Thiel C. M., Zilles K. & Fink G. R. Nicotine modulates reorienting of visuospatial attention and neural activity in human parietal cortex. Neuropsychopharmacology 30, 810–820 (2005). 15668726 10.1038/sj.npp.1300633

[b35] Roy-Byrne P., Radant A., Wingerson D. & Cowley D. S. Human oculomotor function: reliability and diurnal variation. Biol. Psychiatry 38, 92–97 (1995). 7578655 10.1016/0006-3223(94)00225-R

[b36] Hutton S. B. . Smooth pursuit and saccadic abnormalities in first-episode schizophrenia. Psychol. Med. 28, 685–692 (1998). 9626724 10.1017/s0033291798006722

[b37] Ettinger U. . Reliability of smooth pursuit, fixation, and saccadic eye movements. Psychophysiology 40, 620–628 (2003). 14570169 10.1111/1469-8986.00063

[b38] Allman A.-A., Ettinger U., Joober R. & O’Driscoll G. A. Effects of methylphenidate on basic and higher-order oculomotor functions. J. Psychopharmacol. 26, 1471–1479 (2012). 22588495 10.1177/0269881112446531

[b39] Benjamini Y., Drai D., Elmer G., Kafkafi N. & Golani I. Controlling the false discovery rate in behavior genetics research. Behav. Brain Res. 125, 279–284 (2001). 11682119 10.1016/s0166-4328(01)00297-2

[b40] Fisone G., Borgkvist A. & Usiello A. Caffeine as a psychomotor stimulant: mechanism of action. Cell. Mol. Life Sci. 61, 857–872 (2004). 15095008 10.1007/s00018-003-3269-3 PMC11138593

[b41] Curthoys I. S. Generation of the quick phase of horizontal vestibular nystagmus. Exp. Brain Res. 143, 397–405 (2002). 11914784 10.1007/s00221-002-1022-z

[b42] Kashou N. H. . Instruction dependent activation during optokinetic nystagmus (OKN) stimulation: an fMRI study at 3T. Brain Res. 1336, 10–21 (2010). 20403339 10.1016/j.brainres.2010.04.017

[b43] McDowell J. E., Dyckman K. A., Austin B. P. & Clementz B. A. Neurophysiology and neuroanatomy of reflexive and volitional saccades: evidence from studies of humans. Brain Cogn. 68, 255–270 (2008). 18835656 10.1016/j.bandc.2008.08.016 PMC2614688

[b44] Nakamura K. & Hikosaka O. Role of dopamine in the primate caudate nucleus in reward modulation of saccades. J. Neurosci. 26, 5360–5369 (2006). 16707788 10.1523/JNEUROSCI.4853-05.2006 PMC6675290

[b45] Kori A. . Eye movements in monkeys with local dopamine depletion in the caudate nucleus. II. Deficits in voluntary saccades. J. Neurosci. 15, 928–941 (1995). 7823190 10.1523/JNEUROSCI.15-01-00928.1995 PMC6578280

[b46] Hyman S. E. & Holtzman D. M. Molecular neuropharmacology: a foundation for clinical neuroscience. Third edn, (New York: The McGraw-Hill Companies, Inc. 2015, 2014).

[b47] Dorris M. C., Pare M. & Munoz D. P. Neuronal activity in monkey superior colliculus related to the initiation of saccadic eye movements. J. Neurosci. 17, 8566–8579 (1997). 9334428 10.1523/JNEUROSCI.17-21-08566.1997 PMC6573744

[b48] Dorris M. C. & Munoz D. P. A neural correlate for the gap effect on saccadic reaction times in monkey. J. Neurophysiol. 73, 2558–2562 (1995). 7666161 10.1152/jn.1995.73.6.2558

[b49] Everling S. & Munoz D. P. Neuronal correlates for preparatory set associated with pro-saccades and anti-saccades in the primate frontal eye field. J. Neurosci. 20, 387–400 (2000). 10627615 10.1523/JNEUROSCI.20-01-00387.2000 PMC6774131

[b50] Bell A. H., Everling S. & Munoz D. P. Influence of stimulus eccentricity and direction on characteristics of pro- and antisaccades in non-human primates. J. Neurophysiol. 84, 2595–2604 (2000). 11068001 10.1152/jn.2000.84.5.2595

[b51] Reuter-Lorenz P. A., Hughes H. C. & Fendrich R. The reduction of saccadic latency by prior offset of the fixation point: an analysis of the gap effect. Percept. Psychophys. 49, 167–175 (1991). 2017353 10.3758/bf03205036

[b52] Hutton S. B. Cognitive control of saccadic eye movements. Brain Cogn. 68, 327–340 (2008). 19028265 10.1016/j.bandc.2008.08.021

[b53] Nybo L. & Secher N. H. Cerebral perturbations provoked by prolonged exercise. Prog. Neurobiol. 72, 223–261 (2004). 15142684 10.1016/j.pneurobio.2004.03.005

[b54] Backhouse S. H., Biddle S. J., Bishop N. C. & Williams C. Caffeine ingestion, affect and perceived exertion during prolonged cycling. Appetite 57, 247–252 (2011). 21605608 10.1016/j.appet.2011.05.304

[b55] Johnston K. & Everling S. Neurophysiology and neuroanatomy of reflexive and voluntary saccades in non-human primates. Brain Cogn. 68, 271–283 (2008). 18940273 10.1016/j.bandc.2008.08.017

[b56] Thier P. & Ilg U. J. The neural basis of smooth-pursuit eye movements. Curr. Opin. Neurobiol. 15, 645–652 (2005). 16271460 10.1016/j.conb.2005.10.013

[b57] Lencer R. & Trillenberg P. Neurophysiology and neuroanatomy of smooth pursuit in humans. Brain Cogn. 68, 219–228 (2008). 18835076 10.1016/j.bandc.2008.08.013

[b58] Rucker J. C. in *Handbook of Clinical Neurophysiology* Vol. 9 (eds Scott Eggers, D. Z. & David Zee, S.) 18–42 (Elsevier, 2010).

